# An Optimized Method for Single Cell Cloning of Human CAR-T Cells Based on FBS-Coated Plates

**DOI:** 10.34172/apb.43798

**Published:** 2024-12-26

**Authors:** Mahdie Jafari, Shahriyar Abdoli, Masoud Moghaddam Pour, Mohammad Ali Shokrgozar, Zahra Sharifzadeh

**Affiliations:** ^1^Department of Immunology, Pasteur Institute of Iran, Tehran, Iran; ^2^School of Advanced Medical Technologies, Golestan University of Medical Sciences, Gorgan, Iran; ^3^Poultry Viral Vaccines Research and Production Department, Razi Vaccine and Serum Research Institute, Agricultural Research, Education and Extension Organization, Karaj, Iran; ^4^National Cell Bank of Iran, Pasteur Institute of Iran, Tehran, Iran

**Keywords:** Cancer immunotherapy, CAR-T cells, Clonal expansion, 2D culture system, FBS coating, Single cell isolation

## Abstract

**Purpose:**

T cell-based immunotherapy, especially chimeric antigen receptor (CAR)-T cells, has emerged as an appropriate approach for treating hematologic malignancies and is currently under investigation in clinical trials for solid tumors. Despite significant improvements in CAR-T cell production processes, the isolation and expansion of CAR-engineered T cells continue to pose significant challenges. The aim of this research is to provide a simple and cost-effective method for the isolation and expansion of human CAR-T cells. This novel concept applies coated fetal bovine serum (FBS) culture plates and focuses on enhancing viability and functionality to improve the adherence of suspended T cells.

**Methods:**

This study evaluated a two-dimensional (2D) culture technique for isolating the CAR-T cells that target prostate-specific membrane antigen (PSMA) utilizing matrices pre-coated with 0.2% glutaraldehyde and FBS. Jurkat cells were transduced with a lentiviral vector encoding the anti-PSMA CAR construct. FBS-coated and commercialized Matrigel-coated matrices were used for single-cell isolation and clonal expansion. Functional tests were conducted to assess the activation and proliferation of CAR-T cells and the IFN-γ release assay subsequent to cloning and expansion.

**Results:**

Transfection efficiency markedly improved, with 88.4% of Lenti-X 293T cells demonstrating green fluorescent protein (GFP) expression. Among the Jurkat cells, 57.1% showed GFP expression post-transduction, of which 34.1% showed surface expression of anti-PSMA CAR. Clonal expansion on the FBS-coated matrix proved effective, yielding 92.1% GFP-positive isolated cells. Functional assays demonstrated that CAR-T cells co-cultured with LNCaP cells exhibited significantly enhanced proliferation, activation (as indicated by CD69 and CD25 expression), and cytokine release assay (IFN-γ) compared with those co-cultured with DU 145 and mock cells.

**Conclusion:**

This new approach is efficient, economical, and scalable for isolating specific homogenous T cells and promoting their clonal proliferation and expansion. Furthermore, this method improves T cell adherence, proliferation, and functional effectiveness, offering a potential foundation for advancing CAR-T cell therapies aimed at solid tumors. Future research should concentrate on optimizing culture conditions and testing this method in preclinical animal models to ensure its clinical applicability and efficacy.

## Introduction

 Adoptive T-cell treatment has proved promising in cancer immunotherapy through gene-modified T cells that express a tumor-specific T-cell receptor, also known as chimeric antigen receptor (CAR)-T cells.^1^ This therapeutic approach is effective against B-cell malignancies; however, its efficacy in treating solid tumors has not yet been established. Clinical trials with T-cell therapy have demonstrated promising outcomes in the treatment of solid tumors; consequently, researchers have focused on enhancing the reliability and applicability of such therapeutic approaches in this field.^2-4^

 Despite the promising clinical outcomes of CAR-T cell therapies, T-cell heterogeneity remains a significant challenge for the development of effective T-cell-based treatments.^5^ Due to heterogeneity of peripheral blood T lymphocytes, it might be challenging to identify a specific function in a specified T cell population across several types of experiments and obtain clear results.^6^ The pivotal step in adoptive cell therapy is the single-cell isolation and clonal expansion of human CAR-T cells. In a culture of suspended cells, isolating and separating individual cells remains a complex technological problem,^5^ with the yield, purity, and viability of T cells as the key challenges.^7^ Accordingly, developing effective culture methods for these cells is critical for advancing research and clinical study.^8^ Various strategies, including extracellular matrices (ECMs) in both two-dimensional (2D) and three-dimensional (3D) culture techniques, are being explored to enhance the efficiency of single-cell isolation and clonal expansion by improving cell adhesiveness and suspension cell growth.^9^

 Matrigel is widely used among scaffold-based 3D cell culture technologies because of its functional capacity to mimic the ECM.^9-14^ The 3D cell culture technologies are often too complex and expensive, and require specifically trained technical operators, which limits their application to certain research centers. Moreover, particular equipment and skills needed for implementing and sustaining 3D cultures, and the complexity of handling cells in a 3D matrix pose technological obstacles that may limit repeatability.^15^ On the other hand, ongoing investigations to develop more effective 2D culture systems may enable the efficient clonal expansion of single T-cells. Since 2D cultures are simpler to set up and maintain, and require low-cost materials and equipment, they are suitable for large-scale research and laboratory operations.^12,16^ Fetal bovine serum (FBS) mimics the key aspects of the ECM while enhancing cell adhesion, viability, and proliferation.^15,17,18^ The abundance of fibronectin and vitronectin in FBS enhances cell adhesion and proliferation. FBS-coating of cell culture plates significantly improves the growth and function of suspended cells.^8,15,19^ The current study investigates the development of an easy-to-use 2D cell culture method for CAR-T cell isolation and expansion. This paper aims to investigate if FBS coating on surface plates improves the effectiveness of clonal expansion of single suspension cells, and to create a 2D model system for expanding CAR-T cells. After expanding the selected clone, we evaluated the functionality and effectiveness of this second-generation nanobody-based CAR-T cell, which targets prostate-specific membrane antigen (PSMA). This evaluation focused on the expression of the activation markers on the cell surface and the cell’s proliferative capacity against the LNCaP cell line, used as a model for prostate cancer.

## Materials and Methods

###  Cell lines and cell culture conditions

 All cell lines used in this study were purchased from the National Cell Bank of Iran (NCBI), Pasteur Institute of Iran. We grew Jurkat E6.1 cells in RPMI-1640 (Biosera, France) supplemented with 10% FBS (Gibco, Life Technologies, USA), penicillin (100 IU/mL; Sigma-Aldrich), and streptomycin (100 µg/mL; Sigma-Aldrich). Lenti-X 293T, human embryonic kidney 293T (HEK-293T), LNCaP (PSMA-positive human prostate cancer (PC) cell line), and DU 145 (PSMA-negative human PC cell line) were cultured in high-glucose Dulbecco’s modified eagles medium (DMEM; Biosera, France) supplemented with 10% FBS, penicillin (100 IU/mL), and streptomycin (100 µg/mL). Lenti-X 293T and HEK293T cells were used for the production of lentiviral particles and the titration of the produced lentiviruses, respectively. All cell lines were cultured in 95% humidity at 37 °C and 5% CO_2_.

###  CAR construct

 PSMA-NB, a single-domain antibody fragment (nanobody) against PSMA, was used to confer tumor-antigen specificity ((kindly provided by Dr. W. M. van Weerden) in the CAR construct. The second-generation anti-PSMA CAR, comprised of PSMA-NB, CD8α hinge, CD8 transmembrane, 4-1BB endodomain, and CD3-zeta signaling domain was synthesized by Biomatik Company (Cambridge, Canada) and subcloned into a lentiviral expression vector pCDH under the CMV promotor. The plasmid was then propagated in *Escherichia coli* (DH5α) and confirmed through colony PCR, restriction enzyme confirmation, and Sanger sequencing.

###  Lentivirus production 

 To generate the lentivirus, 4 × 10^6^ Lenti-X 293T cells were seeded into a 6-cm cell culture petri dish 4-5 hours prior to transfection in DMEM with 10% FBS. Lenti-X293T cells were transfected with pCDH transgene plasmid, packaging plasmid (psPAX2), and envelope plasmid (pMD2.G) using a mix of polyethyleneimine (PEI) and serum-free media (DMEM). At 6 hours post-transfection, the medium was replaced with fresh medium. After 24 h, the supernatant containing the lentiviral particles was collected in conical tubes every 12 h until 48 h post-transfection and stored at 4 °C. Flow cytometry was used to evaluate the transfection efficacy in the Lenti-X 293T cell line after 72 hours. To this end, green fluorescent protein (GFP) expression was measured by flow cytometry as a hallmark of CAR expression (Partec PAS-III).

###  Lentivirus titration

 Different amounts (10, 20, 50, 100 and 250 µL) of the harvested virus were used to transduce HEK-293T cells, according to the Trono Lab protocol.^20^ In brief, the amounts of crude virus were mixed with 5 × 10^4^ HEK-293T cells and raised to 1 mL with complete DMEM. After 48 h, the percentage of GFP-positive cells was measured by flow cytometry. Viral titer (transducing units (TU)/mL) were calculated as follows:


$$Titer\left(\frac{TU}{mL}\right)=\frac{Total\;number\;of\;seeded\;cells\times \left[\frac{\%\;of\;GFP-positive\;cells}{100}\right]}{Volume\;of\;non-concenstrated\;vector}$$

After the calculation of virus titer, the multiplicity of infection (MOI) was also determined as follows:


$$MOI=\frac{viral\;particles\;used\;per\;well}{number\;of\;cells\;originally\;seeded}.$$

###  Jurkat cell transduction 

 To express the CAR construct in Jurkat cells, 1 × 10^5^ cells were transduced with MOI = 20 in complete RPMI-1640 in a 24-well plate. Flow cytometry was used to identify GFP-positive cells, as the indicator of CAR gene expression, after incubating cells for 72 hours.

###  Evaluation of the surface expression of CAR

 To evaluate surface expression of anti-PSMA-CAR, 1 × 10^6^ transduced cells were resuspended in 100 μL staining buffer (phosphate-buffered saline (PBS) supplemented with 2% FBS). Then, 0.5 μg MonoRab^TM^ rabbit anti-Camelid V_HH_ antibody (GenScript, China) and 0.5 μg PE-donkey anti-rabbit IgG secondary antibody (BioLegend, USA) were added in sequence, and incubated for 45 minutes at 4 °C. After washing the cells with PBS, flow cytometry was performed.

###  Limiting dilution and single-cell isolation

 Single anti PSMA CAR-T cells were isolated by serial dilution and seeded in 96-well plates. Then, 10 mL of a 10 cell/mL cell suspensions was prepared, 100 µL of that cell solution was transferred into each well of six 96-well plates and incubated at 37 °C in 5% CO2 for 10–14 days. All wells were examined for two weeks and the single clones were transferred to a new well and propagated.

###  Matrix preparation 

 For FBS exposure, 96-well treated cell culture plates (SPL, Korea) were pre-coated with 0.2% glutaraldehyde for 15 minutes to activate the hydroxyl groups of the treated surface plate, and were washed three times with deionized water. Next, 300 µL of FBS was added to plates coated with dry glutaraldehyde and left at 56 °C for 30 minutes, and then air-dried at room temperature for 24 hours.^21,22^ Matrigel-coated plates serving as the positive control were prepared as follow: 40 µL of cold Corning® Matrigel® Matrix (Corning, USA) was added to each well of a 96-well plate and solidified at 37 °C for 45 minutes. For FBS/Matrigel-coated matrix preparation, first, FBS and Matrigel were coated, respectively, as mentioned above. Then, a 10 cells/mL cell suspension was prepared for isolating the single anti-PSMA-CAR-T cells by serial dilution. Then, 100 µL of the cell solution was transferred into each well of prepared FBS-coated, Matrigel-coated, and FBS/Matrigel-coated 96-well plates and incubated at 37 °C in 5% CO_2_. Until the formation of colonies, the fresh medium was added during expansion. After clonal expansion, cells were transferred into 24-well plates and proliferated in complete RPMI-1640 at 37 °C in 5% CO_2 _for further analysis.

###  CAR-T cell activation assay

 In a 96-well tissue culture plate, 10^3^ target cells (LNCaP and DU 145) were seeded and co-cultured with anti-PSMA-CAR-T cells with an effector-to-target (E:T) ratio of 3:1. Anti-PSMA-CAR-T cells were harvested after 18 and 24 h then washed with PBS. 1 × 10^6^ cells were resuspended in 100 µL staining buffer containing 0.25 μg PE‐conjugated anti‐human CD69 and PE- anti human CD25 antibody (BioLegend, USA), incubated at 4 °C for 45 minutes. Then, the target and mock T (Transduced pCDH) cells were analyzed by flow cytometry.

###  CAR-T cell proliferation assay

 MTT (3-[4,5-dimethylthiazol-2-yl]-2,5-diphenyl tetrazolium bromide) assay was performed to evaluate the proliferation rate of anti-PSMA-CAR-T cells co-cultured with target cells. To this end, 3 × 10^4^ target cells were seeded per well in 100 μL of complete DMEM and incubated at 37 °C for 16 hours. Then, anti-PSMA-CAR-T cells were co-cultured with target cells in an E:T ratio of 10:1 and 3:1 for 48 hours. Next, 96-well plates were centrifuged at 500 × g for 10 minutes, and the supernatant was gently removed. After that, 20 μL of MTT (Sigma-Aldrich, USA) solution (5 mg/mL in PBS) was added to each well and incubated at 37 °C for 4 hours. Then, formazan crystals were dissolved in 150 μL of DMSO, and optical density was measured at 590 nm. The proliferation rate was measured as follows:


$$\frac{OD\left(experimentalwell-tumor\;cells\;without\;effector\right)}{OD\left(corresponding\;number\;of\;effector\;cells-media\;\right)}\times 100$$

###  Cytokine release assay

 A 96-well tissue culture plate was inoculated with 2 × 10¹ target cells per well, namely LNCaP and DU 145. After 16 hours of incubation, E:T ratios of 1:10 (2 × 10^2^ cells/well) and 3:1 (6 × 10⁴ cells/well) were used to compare the two CAR-T and mock T cells groups to evaluate interferon γ (IFN‐γ) secretion, the supernatant was harvested after 24 hours and evaluated by an ELISA kit (Quantikine Kit; R&D Systems, Minneapolis, MN). The standard curve was utilized to determine the concentrations.

###  S tatistical analysis

 Data was analyzed in GraphPad Prism software version 8.0 using repeated measures two-way ANOVA test where appropriate. Mean relative value ± standard deviation was used to present the number of T cells expansion after coculturing with targeted cell lines and the percentage of CAR-T cells. The results are shown as percentage in histograms.

## Results

###  Lenti -X 293T cell transfection and virus production

**Figure 1:**
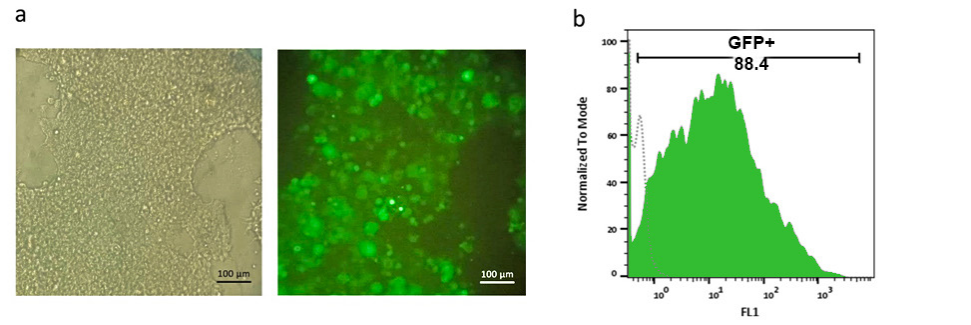
Lenti-X 293T cell transfection and GFP expression analysis.a. Phase contrast (left) and fluorescence microscopy (right) images of Lenti-X 293T cells transfected with the pCDH-anti-PSMACAR construct, where GFP expression is clear. Scale bar: 100 µm. b. Flow cytometry histogram showing GFP expression in Lenti-X 293T cells, where 88.4% of the cells are GFP-positive, indicating a high transfection efficiency

 Lenti-X 293T cells were transfected with pCDH-anti-PSMA-CAR, psPAX2, and pMD2.G to produce lentiviral vectors. After 48 hours, 88.4% of Lenti-X cells expressed GFP as the reporter protein (Figures 1a and 1b).

###  Lentivirus titration 

**Figure 2:**
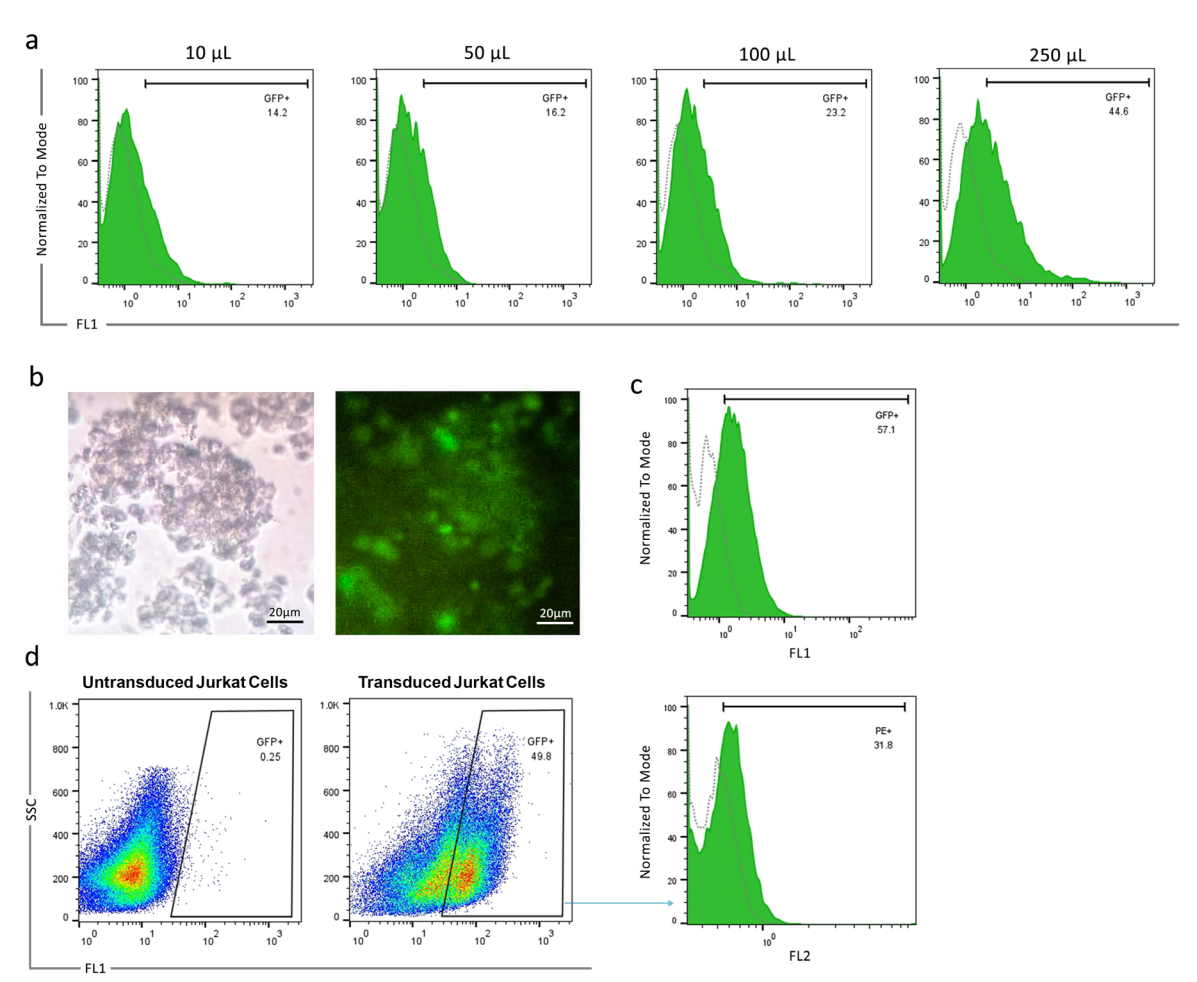
Lentivirus titration and CAR expression in transduced Jurkat cells.a. Flow cytometry analysis of GFP expression in HEK-293T cells transduced with different amounts (10 µL, 50 µL, 100 µL, and 250 µL) of harvested lentiviral supernatant. The GFP-positive rates were 14.2%, 16.2%, 23.2%, and 44.6%, respectively. b.Phase contrast (left) and fluorescence microscopy (right) images of Jurkat cells transduced with the lentiviral vector expressing GFP. Scale bar: 20 µm. c. Flow cytometry histogram showing GFP expression in 57.1% of Jurkat cells following transduction. d. Flow cytometry histogram showing surface expression of anti-PSMA-CAR in 31.8% of engineered Jurkat cells

 To assess the titration of lentiviral vectors, HEK-293T cells were transduced with varying concentrations of crude supernatant. Our results showed that 10, 50, 100, and 250 µL of crude supernatant of the harvested virus corresponded to 14.2%, 16.2%, 23.2%, and 44.6% transduction rates, respectively (Figure 2a). Then, 10 µL of crude supernatant was considered for viral titration. The viral titration was calculated at 1.03 × 10^7^ TU/mL.

###  Transduction of Jurkat cells and evaluation of CAR surface expression 

 Jurkat cells were transduced with a lentiviral vector at a MOI = 20. Following efficient transduction, fluorescent microscope analysis revealed GFP-positive cells. Flow cytometry determined that 57.1% of Jurkat cells express GFP (Figures 2b and 2c). Next, the anti-PSMA-CAR expression was evaluated among the GFP-positive Jurkat cell population. The results showed that anti-VHH antibodies are capable of detecting anti-PSMA-CAR constructs on the surface of 31.8% of GFP-positive Jurkat cells (Figure 2d).

###  Single-cell isolation on different matrices and clonal expansion 

**Figure 3:**
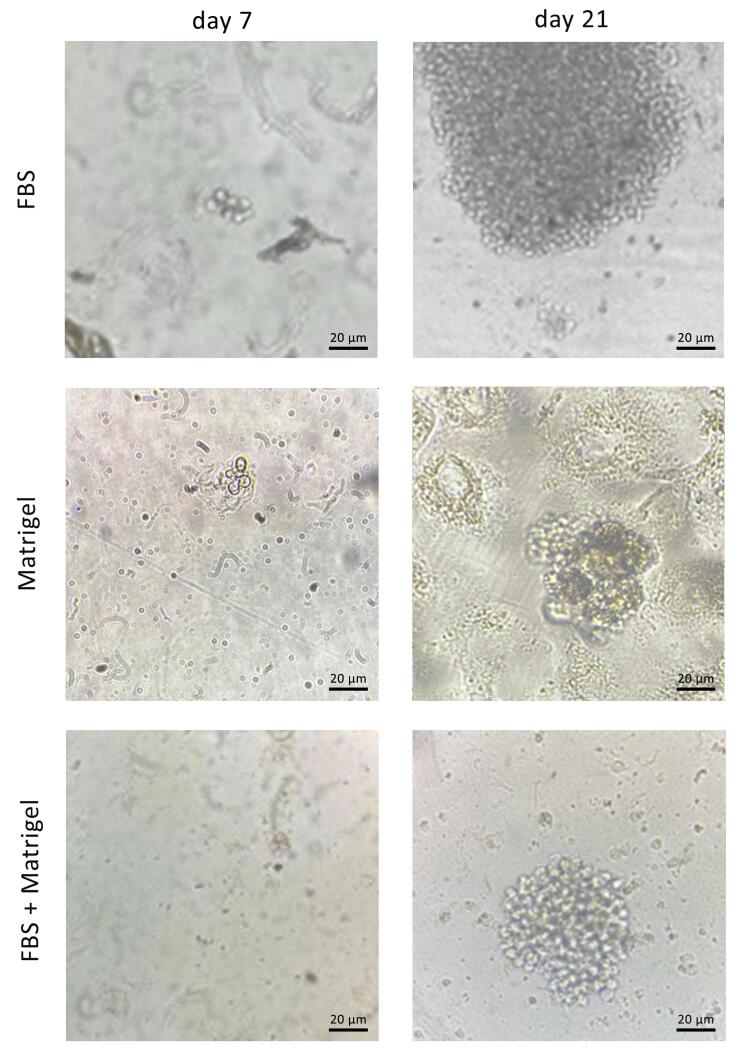
Single-cell isolation and colony formation of anti-PSMA-CAR-T cells. Phase contrast images of the anti-PSMA-CART cells on. a. FBS-coated, b. Matrigel-coated, and c. FBS + Matrigel-coated matrices at day 14 (left) and day 21 (right). Scale bar: 20 µm

**Figure 4:**
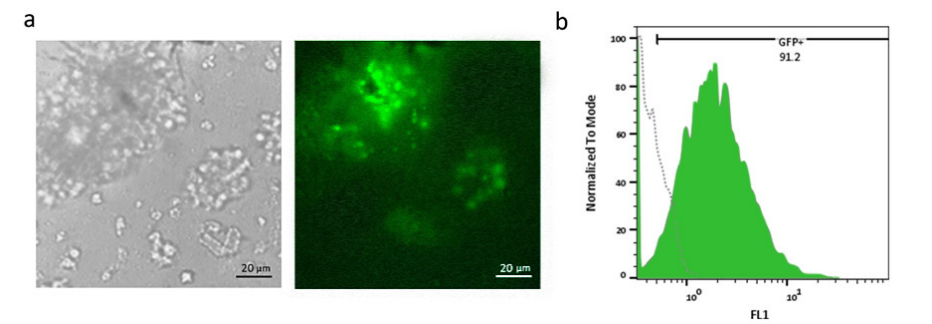
Transduction efficiency and GFP expression analysis in Jurkat cells.a. Phase contrast (left) and fluorescence microscopy (right) images of Jurkat cells transduced with lentiviral vectors expressing GFP. High transduction efficiency is evident from widespread expression of GFP. Scale bar: 20 µm. b. Flow cytometry histogram shows that 91.2% of Jurkat cells are GFP-positive that proves the efficient expansion of isolated Jurkat cells

 After preparation of FBS-coated, Matrigel-coated, and FBS/Matrigel-coated matrices, anti-PSMA-CAR-T cells were subjected to a limitation dilution (1 cell/well). After 14 days, the low-count colonies were monitored and followed for 21 days to promote generation of high-count colonies (Figure 3). We could not separate the colonies on Matrigel-coated and Matrigel/FBS-coated matrices because they were entrapped in 3D matrices. Contrarily, colonies on FBS-coated matrix did not exhibit a strong attachment; therefore, they were separated easily and processed later. Then, the separated cells were transferred into a 24-well plate and expanded until they reached a suitable count for further analysis. After expansion, the purity of the isolated cells was assessed in terms of the expression of the CAR construct. The results showed that 92.1% of isolated cells expressed GFP as an indicator of CAR expression (Figure 4).

###  Activation of anti-PSMA-CAR-T cells 

**Figure 5:**
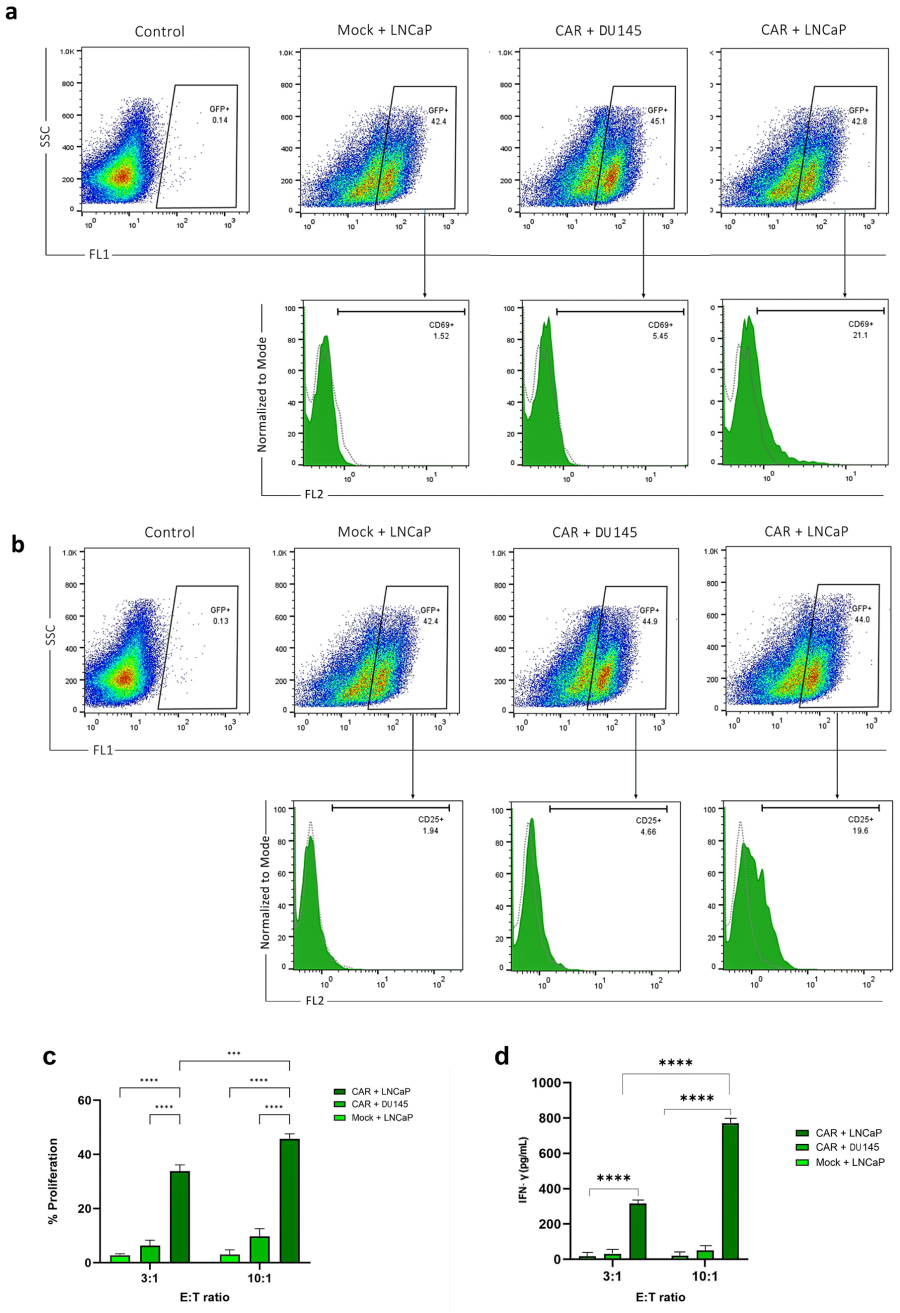
CAR-T cell proliferation, T-cell activation marker and cytokine release assay.a. Flow cytometry histograms show CD69 and b. CD25 expression in anti-PSMA CAR-T cells that are co-cultured with LNCaP cells at left side and with DU 145 cells at the right side. The percentage of CD69 and CD25-positive cells (PE + ) is 21% and 19.6% in the CAR-T cells co-cultured with LNCaP and DU 145 cells and mock cells. c. Proliferation rate of anti-PSMA CAR-T cells that were co-cultured with LNCaP and DU 145 cells at effector-to-target (E) ratios of 3:1 and 10:1. The proliferation rate is significantly high when CAR-T cells are co-cultured with PSMA-positive LNCaP cells compared to PSMA-negative DU 145 cells and mock cells. The statistical significance was represented as *** *P* < 0.001 or **** *P* < 0.0001. d. Anti-PSMA CAR and fake T cells were cocultured with target cells at 3:1 and 10:1 effector-to-target ratios, and after 48 hours, the production of IFN-γ was assessed. T cells were measured using ELISA. Results were presented as means ± SD (**** *P* < 0.0001)

 Surface expression of CD69 and CD25 was considered a T-cell activation marker in the co-culture of anti-PSMA-CAR-T cells with LNCaP, DU 145 and mock cells. Our results showed that 21.1% and 20.2% of anti-PSMA-CAR-T cells co-cultured with LNCaP cells expressed CD69 and CD25 on their surfaces, respectively, those co-cultured with DU 145 and mock cells did not show any surface expression (Figure 5a and 5b).

###  Proliferation of anti-PSMA-CAR-T cells

 MTT assay was run to assess the relative proliferation rate of anti-PSMA-CAR-T cells co-cultured with LNCaP and DU 145 cells in different E:T ratios. Our results showed that LNCaP and DU 145 cells in E:T 3:1 and 10:1 significantly improved proliferation of anti-PSMA-CAR-T cells (*P* value < 0.0001 for both). Furthermore, the proliferation of anti-PSMA-CAR-T cells co-cultured with LNCaP with E:T 10:1 significantly improved than those with E:T 3:1 (*P* value = 0.0002; Figure 5c).

###  Cytokine release assay

 T cells were cocultured with LNCaP and DU 145 cells in E:T ratios of 10:1 and 3:1, respectively, to assess the capacity of anti-PSMA-CAR-T cells to secrete IFN‐γ in reaction to the target cells. The cytokines were tested 24 h later after collecting the supernatant. As seen in Figure 5, when CAR-T cells interacted with LNCaP cells, they released much more IFN-γ than when they interacted with DU 145 cells or mock cells. The amounts were about 900 pg/mL in a 10:1 ratio and 400 pg/mL in a 3:1 ratio (Figure 5d).

## Discussion

 Single-cell isolation is essential for various applications, such as the development of monoclonal stable cell lines, the production of monoclonal antibodies, gene editing, and stem cell and CAR-T cell therapies.^21,22^ As for suspended cells, isolating and separating individual cells remains a complicated technique.^6^

 ECM regulates a diverse array of fundamental processes essential for the growth, operation, and stability of all eukaryotic cells.^23,24^ Matrigel, a commercialized matrix, is widely used for cell culture and expansion.^23-25^ Nonetheless, it has limited use in cellular biology and therapeutic cell manufacture due to its complicated batch-to-batch variation, lack of repeatability, high cost, and safety concerns.^25,26^ Furthermore, Matrigel is not suitable for physical or pharmacological modifications, making it difficult to fine-tune the matrix to promote the desired cell behaviors and accomplish specific biological results.^10,25-27^ Therefore, are actively pursuing the development of straightforward and effective methods for single-clone isolation.

 Currently, a heterogeneous population of transduced CAR-T cells and non-transduced T cells is transferred to patients. The efficacy of CAR-T therapies can be significantly influenced by this heterogeneity. Consequently, additional research is required to enhance the efficiency of CAR-T cell manufacturing methodologies. The optimal function and expansion of these cells are not achieved when they are expanded under complex and suboptimal conditions. This can impede their capacity to face the immunosuppressive and challenging conditions that surround solid tumors, potentially resulting in T cell exhaustion and a loss of functionality.^28,29^ On the other hand, CAR-T cells are more likely to be able to survive in the immunosuppressive tumor microenvironment after being infused if they are grown in conditions that help them multiply and spread out evenly. This is due to the fact that T cells that are cultured in optimal conditions with nutrient-rich media are clonally expanded, resulting in increased survival and functional capacity.^30^ Consequently, they have a better ability to respond to the tumor microenvironment. Biomaterials derived from naturally occurring ECM have generated remarkable interest in tissue engineering and regenerative medicine.^27,31,32^ Numerous studies have demonstrated that cell adhesion in anchorage-dependent cells is mediated *in vitro* by the adsorption of serum proteins in the growth medium.^9^ Johannes Hackethal described several ways for isolating human ECM components from diverse sources and found that active serum recovered from ECM had significantly lower DNA remains than the Tris-NaCl separation technique. However, due to the complex composition of the serum, which contains numerous bioactive proteins, the precise methods of decellularization remain unidentified and may depend on many pathways.^33^ Rainaldi et al investigated the role of fibronectin, vitronectin, and other ECM proteins in the adhesion of K562 cells to the positively charged polylysine surface. Their findings indicated that cells on FBS-coated plates exhibited prolonged survival and maintained their morphology.^34^

 According to Zhang et al, cells in two-dimensional cultures are polarized such that only a portion of their membrane may communicate with the ECM and other cells in the culture. The remaining portions of the cell are exposed to the bulk culture fluid.^35^ In 2008, Gieni and Hendzel discovered that this process leads to mechanical transfer and polarized integrin binding, which are not normal. In two-dimensional cultures, cells are exposed to the same uniform concentration of growth factors, cytokines, nutrients, and membrane-bound cytokines as in the bulk medium. Soluble factors that affect cell motility, cell-cell communication, and differentiation have dynamic concentration gradients that fluctuate over time within living organisms.^36^

 FBS has long been extensively used as a crucial component in cell expansion media, largely owing to its capacity to promote cell adhesion and support cellular proliferation.^34^ Researchers compared the development and differentiation of primary human bone marrow MSCs (BMSCs) grown in conventional FBS-containing αMEM medium to a commercial serum-free medium. The FBS-containing media was supplemented with Fibroblast Growth Factor-2 (FGF2), which increases cell proliferation and maintains a stem-like state.^16,28^ Abbasalipour et al developed a simple and effective approach for enhancing the efficacy of lentiviral transduction in K562 cells by promoting cell adherence to the plate surface using FBS. The flow cytometry revealed that the transduction rate in K562 cells reached 64.5% following treatment with FBS on the plate.^37^

 Scientists developed anti-CD3 (aCD3) nanoarrays, a new platform for stimulating T cells which are made using site-directed protein immobilization and self-assembled nanopatterning, and provide precise control of ligand orientation and surface density. ACD3 density on nanoarrays is linked to the activation of primary human CD4 + T lymphocytes, as demonstrated by CD69 expression, IL-2 production, and cell growth. Nanopatterning facilitated the immobilization of aCD3, which led to an unprecedented finetuning of the T cell response, and a much higher rate of cell activation on these surfaces than that on aCD3-coated plastics.^37^

 This study aimed to develop an optimized, straightforward, and economical method for isolating human T cells, while preserving their capacity for expansion and functionality. A second-generation nanobody-based CAR-T cell targeting PSMA antigen was expressed in Jurkat T cells using lentivirus vectors. Single CAR-T cells were effectively isolated and expanded using an optimized FBS-based method. Functional testing showed that the expanded CAR-T cells could recognize and target PSMA-expressing LNCaP prostate cancer cells. Increased CD69 expression in effector cells co-cultured with target cells confirmed the functional integrity and specificity of CAR-T cells.^38^ Johnson et al observed that activation marker expression predicts CAR-T cell efficacy, which corroborates our findings.^39^ The proliferation assay demonstrated that the expanded single CAR-T cell clone exhibited significantly greater proliferation when cocultured with target cells, compared to control cells. In 2016, antigen-expressing target cells were found to boost CAR-T cell proliferation and cytotoxicity.

 Our 2D culture technique with an FBS-coated matrix is an economical and accessible approach for therapeutic use. This approach corroborates the previous studies indicating that mimicking the *in vivo* ECMs improves cell expansion. Nevertheless, in comparison to more conventional 2D cultures, 3D systems are more accurate representations of the intricate architecture and relationships observed in nature. Cells cultured in 3D environments experience mechanical signals that more accurately mimic physiological conditions, along with varying amounts of oxygen, nutrients, and growth agents.^40^ All of these factors have a major impact on how cells function, from proliferation to migration to medication responsiveness. Improved 3D culture techniques, such as organoids and custom ECMs, have expanded the horizons for studying developmental and pathological events, elucidating molecular pathways, and potentially enhancing drug discovery research. This method, which mimics *in vivo* conditions, is however not appropriate for isolating T cells due to challenges in enhancing viability and isolating Matrigel.^10^

 Our functional experiments demonstrated that CAR-T cells can proliferate. This finding is similar to what Jia et al reported, i.e., fibronectin coatings boosted T cell proliferation and function and were activated against PSMA-positive LNCaP cells more than DU 145 cells.^12,41^ Ghassemi et al also found that CAR-T cell expansion in a concentrated growth factor medium improved engraftment and effectiveness.^42^

 Nonetheless, long-term challenges remain. T cell behavior is very variable, and the CAR-T cell population’s natural heterogeneity may increase this variability further over time. This variation may affect the expansion uniformity and repeatability as well.^5,10^ Even though 2D culture may be a simple and economical way for cell isolation and expansion, setting up *in vivo* is necessary. Future research should concentrate on hybrid systems that combine the benefits of simple and affordable 2D cultures with the physiological significance of 3D matrices. Future studies are recommended to compare the functional assays of CAR-T cells in a 3D platform with those in a 2D platform. Modern imaging and single-cell analysis capabilities might help optimize culture conditions by revealing CAR-T cell interactions with cancer cells. Moreover, to translate these insights into therapeutic applications, our enhanced 2D culturing approach must undergo testing in preclinical settings.

## Conclusion

 In conclusion, our findings contribute to a feasible strategy for enhancing the clonal growth of CAR-T cells using the improved 2D culture procedures. This technique, which addresses the scalability and efficacy challenges, has the potential to significantly accelerate the development and implementation of CAR-T cell therapy, especially in solid tumors. By promoting cell proliferation under conditions that enhance viability and utilizing FBS as a matrix for adhesion and proliferation, we anticipate that the cells will exhibit enhanced performance and power when confronted with a cancerous cell. Furthermore, the 3D environment may be employed afterwards and in the clinic to maintain and improve the environment of the target cells and research should focus on confirmation of these results and the *in vivo* testing of CAR-T cells to ensure their functionality and therapeutic potential.

## Competing Interests

 The authors declare that they have no conflict of interest.

## Ethical Approval

 All experiments have been conducted according to the guidelines of the Ethics Committee of the Pasteur Institute of Iran (PII), IR.PII.REC.1398.009.
